#  Synthesis, Characterization and *In vitro* Antitumour Activity of Novel Organotin Derivatives of 1,2- and 1,7-Dicarba-*Closo*-dodecaboranes

**DOI:** 10.1155/MBD.1995.37

**Published:** 1995

**Authors:** Marcel Gielen, François Kayser, Olga B. Zhidkova, Vladimir Ts. Kampel, Vladimir l. Bregadze, Dick de Vos, Monique Biesemans, Bernard Mahieu, Rudolph Willem

**Affiliations:** 1 Free University of Brussels, VUB Department of General and Organic Chemistry Faculty of Engineering Room 8G512 Brussels Belgium; 2 Room 8G512 High Resolution NMR Centre, HNMR Pleinlaan 2 Brussels B-1050 Belgium; 3 A.N. Nesmeyanov Institute of Organoelement Compounds Russian Academy of Sciences Moscow 117813 Russia; 4 Pharmachemie BV Medical Department Haarlem NL-2003 RN Netherlands Antilles; 5 Catholic University of Louvain INAN Louvain-la-Neuve Belgium

## Abstract

Several organotin derivatives of 1,2- and 1,7-dicarba-*closo*-dodecaboranes were synthesized and characterized by ^119^Sn Mössbauer, ^1^H, ^13^C and ^119^Sn NMR spectroscopy. Their antitumour activities *in vitro* against cancerous cell lines of human origin are reported.


### SYNTHESIS, CHARACTERIZATION AND *IN VITRO* ANTITUMOUR ACTIVITY OF NOVEL ORGANOTIN DERIVATIVES OF 1,2- AND 1,7-DICARBA-*CLOSO*-DODECABORANES

Marcel Gielen[1*], François Kayser[1,2], , Olga B. Zhidkova[3], Vladimir Ts. Kampel[3], Vladimir I. Bregadze[3], Dick de Vos[4], Monique Biesemans[1,2], Bernard Mahieu[5], and Rudolph Willem[1,2]

Free University of Brussels, VUB
[1*] Department of General and Organic Chemistry of the Faculty of Engineering, Room 8G512, and
[2] High Resolution NMR Centre, HNMR, Pleinlaan 2, B-1050 Brussels, Belgium.
[3] A.N. Nesmeyanov Institute of Organoelement Compounds, Russian Academy of Sciences, Moscow 117813, Russia.
[4] Pharmachemie BV, Medical Department, NL-2003 RN Haarlem, Netherlands.
[5] Catholic University of Louvain, INAN, Louvain-la-Neuve, Belgium

### Abstract

Several organotin derivatives of 1,2- and 1,7-dicarba-*closo*-dodecaboranes were synthesized and characterized by $^{119}Sn$ Mössbauer, $^{1}H$, $^{13}C$ and $^{119}Sn$ NMR spectroscopy. Their antitumour activities *in vitro* against cancerous cell lines of human origin are reported.

### Introduction

Boron derivatives present a potential interest in the anticancer therapy by neutron capture[1] provided these compounds exhibit a sufficiently selective affinity towards tumour cells[2]. On the other hand, numerous tin derivatives exhibit promising *in vitro* antitumour activities[3] against as well as selectivities[4] towards some human cancer cell lines. Thus, such favourable properties can be expected from compounds combining boron and tin. In this paper we report the synthesis, characterization and *in vitro* antitumour activities of such compounds derived from 1,2- and 1,7-dicarba-*closo*-dodecaboranes[5] (ortho- and meta-carborane, respectively).

### Synthesis

The ortho- and meta-carboran-9-yltin dichlorides were prepared according to [5]:

$(C_2B_{10}H_{11}\text{-}9)_2Hg + SnCl_2 \rightarrow (C_2B_{10}H_{11}\text{-}9)_2SnCl_2 + Hg.$

The dicarboranyltin dichlorides were transformed into the corresponding oxides by reaction with dilute aqueous sodium hydroxide:

$(C_2B_{10}H_{11}\text{-}9)_2SnCl_2 + 2NaOH \rightarrow (C_2B_{10}H_{11}\text{-}9)_2SnO + H_2O + 2NaCl.$

Both ortho- and meta-carboranyl tin oxides were reacted with 2,6-pyridine dicarboxylic acid in a 1:1 molar ratio under elimination of water.

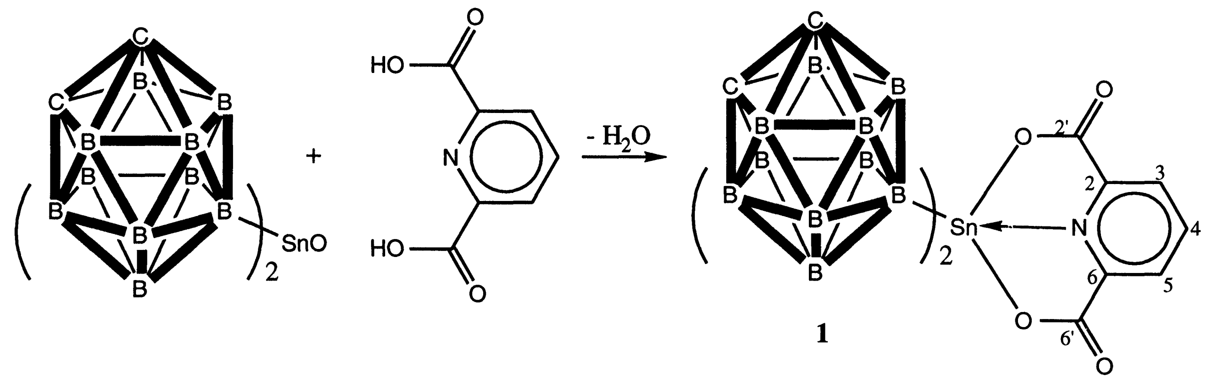

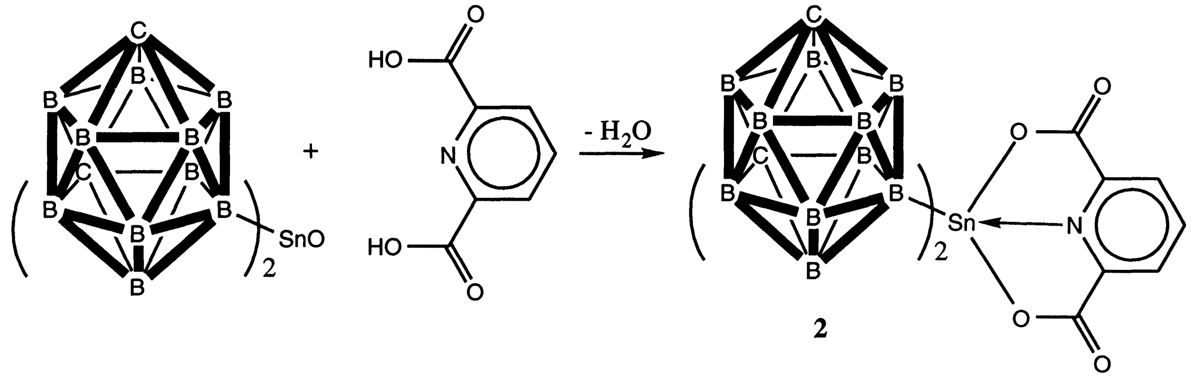

The ortho-carboranyl tin oxide was likewise reacted with 2-L-pyrrolidone-5-carboxylic acid in a 1:2 molar ratio:

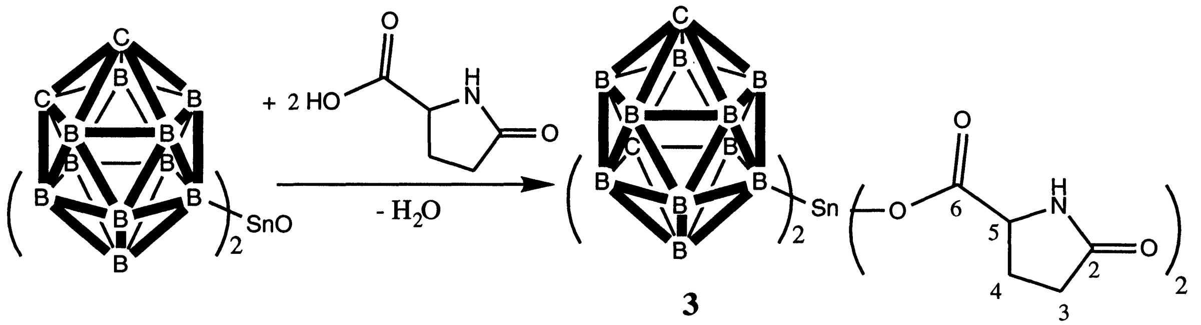

### Antitumour Activities

The *in vitro* antitumour activities observed for different human cancer cell lines are shown in Table 1. *In vivo* screening results are given in table 2.

The high *in vitro* antitumour activity of **1** and **2** are noteworthy, especially for the MCF-7 line, where they are comparable to those of doxorubicin. Bis(metacarboranyl)tin dichloride exhibits even higher activities in both MCF-7 and WiDr of the order of those of doxorubicin. Compound **3** exhibits lower activities, though they remain superior to those of cisplatin.

Compound **2** is active *in vivo* against L1210 murine leukemia at doses of 7 and 10 mg/kg but toxic at 14 mg/kg (table 2). Noteworthy is that one mouse was cured at a dose of 7 mg/kg.

**Table 1.** *In vitro* antitumour activities of compounds **1** to **3** as well as of $o$-$C_2B_{10}H_{12}$ and ($m$-$C_2B_{10}H_{11}$-9)$_2SnCl_2$ against two human cancer cell lines.

| Compounds | MCF-7 | WiDr | EVSAT | IGROV | M19MEL | A498 |
|---|---|---|---|---|---|---|
| $o$-$C_2B_{10}H_{12}$ | 36817 | 22456 | - | - | - | - |
| ($m$-$C_2B_{10}H_{11}$-9)$_2SnCl_2$ | 5 | 31 | - | - | - | - |
| **1** (DMSO) | 10 | 102 | - | - | - | - |
| (EtOH) | 14 | 197 | | | | |
| **2** | 11 | 45 | - | - | - | - |
| **3** | 60 | 410 | 48 | 3 | 30 | 110 |
| Doxorubicin | 8 | 20 | 6 | 28 | 5 | 5 |
| Cisplatin | 800 | 1200 | 650 | 79 | 530 | 1200 |

Activities of **3** against other cell lines: A204: 49; IgR-37: 12; T24: 32

**Table 2.** *In vivo* antitumour screening of compound **2** on groups of male DBA/2 mice bearing leukaemia L1210. Doses applied, mean body weights, median survival times (MST), T/C values and long-term survivors (LTS). The drug doses (mg/kg) were administered i.p. in a single dose.

| | Mean body weight (g) | | | MST | T/C | LTS |
|---|---|---|---|---|---|---|
| Treatment | day 1 | day 5 | day 7 | (day) | % | |
| 7 mg/kg i.p. | 25.6 | 23.4 | 25.3 | 14 | 140 | 1/6 |
| 10 mg/kg i.p. | 25.4 | 21.1 | 21.1 | 14.5 | 145 | 0/6 |
| 14 mg/kg i.p. | 24.6 | 20.7 | 20.4 | 5 | 50 | 0/6 |
| Control | 25.1 | 25.9 | 27.4 | 10 | 100 | 0/9 |

### Experimental Section

### *Instruments and Procedures*

The Mössbauer spectra were recorded as described previously[6]. The Mössbauer parameters (IS towards $Ca^{119}SnO_3$, QS, Γ) are given in mm/s.

The NMR spectra were recorded on a Bruker AC250 and/or a Bruker AMX500 spectrometer. On the AC250 instrument $^{1}H$, $^{13}C$ and $^{119}Sn$ spectra were recorded at 250.13, 62.90 and 93.28 MHz respectively. On the AMX500 spectrometer $^{1}H$ and $^{13}C$ spectra were recorded at 500.13 and 125.76 MHz respectively.

$^{1}H$ and $^{13}C$ chemical shifts were referenced to the residual solvent peaks and converted to the standard TMS scale. The $^{119}Sn$ chemical shift is given with respect to neat external tetramethyltin ($\Xi(^{119}Sn)$ = 37.290665 MHz)[7,8]. Chemical shifts are given in ppm and coupling constants in Hz. Couplings involving the $^{1}H$, $^{117/119}Sn$ and $^{11}B$ nuclei are given between parenthesis, brackets and accolades respectively. Abbreviations used below: b = broad; s = singlet; d = doublet; t = triplet; sep = septet; m = complex pattern.

### *Synthesis and Characterization*

$(o\text{-}C_2B_{10}H_{11}\text{-}9)_2SnCl_2$[5]: Mössbauer: IS = 1.66; QS = 3.49; $\Gamma_1$ = 0.87; $\Gamma_2$ = 0.94.

$(m\text{-}C_2B_{10}H_{11}\text{-}9)_2SnCl_2$[5]: Mössbauer: IS = 1.66; QS = 3.50; $\Gamma_1$ = 0.86; $\Gamma_2$ = 0.86.

$(o\text{-}C_2B_{10}H_{11}\text{-}9)_2SnO$: A solution of 163.0 mg (4.07 mmol) of NaOH in 60 mL of $H_2O$ was added dropwise to a solution of 807.6 mg (1.70 mmol) of $(o\text{-}C_2B_{10}H_{11}\text{-}9)_2SnCl_2$ dissolved in 30 mL diethylether. After 2 hours, the precipitate formed was filtered off under vacuum and washed with water and diethylether. Yield: 77 %. Mössbauer: IS = 1.27; QS = 2.50; $\Gamma_1$ = 1.10; $\Gamma_2$ = 1.09.

$(m\text{-}C_2B_{10}H_{11}\text{-}9)_2SnO$: A similar procedure as for $(o\text{-}C_2B_{10}H_{11}\text{-}9)_2SnO$ was used. Yield: 86 %. Mössbauer: IS = 1.39; QS = 3.27; $\Gamma_1$ = 0.98; $\Gamma_2$ = 0.94.

Compound **1**: 371 mg (0.88 mmol) of $(o\text{-}C_2B_{10}H_{11}\text{-}9)_2SnO$ were added to a solution of 147 mg (0.88 mmol) of 2,6-pyridine dicarboxylic acid in 50 mL of ethanol/toluene (10/40). The mixture was refluxed during 6 hours and the water/ethanol/toluene ternary azeotrope was distilled off with a Dean-Stark funnel. Half the solvent was further evaporated. The white crystals formed after one night were filtered off and recrystallised from ethanol. Yield: 65 %. mp 325-328 °C dec. Mössbauer: IS = 1.56; QS = 3.82; $\Gamma_1$ = 1.00; $\Gamma_2$ = 0.96. NMR (DMSO): $^1H$ NMR: $C_2H_2$: 3.38 (bs); $B_{10}H_9$: 1.1-3.5 (b); H3 = H5: 8.50 (d, 7.6); H4: 8.69 (t, 7.6). $^{13}C$ NMR: $C_2H_2$: 59.8 [39]; C2 = C6: 145.4; C3 = C5: 126.5; C4: 146.7; C2' = C6': 162.5. $^{119}Sn$ NMR: -166.3 {1:2:3:4:3:2:1 sep from couling with $^{11}B$ (I = 3/2), 1268}.

Compound **2**: A similar procedure as for compound **1** was used. Yield: 70%; mp > 350 °C. Mössbauer: IS = 1.52; QS = 3.70; $\Gamma_1$ = 0.91; $\Gamma_2$ = 0.95. NMR (DMSO): $^1H$ NMR: $C_2H_2$: 3.49 (bs), $B_{10}H_9$: 1.0-3.5 (b); aromatic: H3 = H5: 8.50 (d, 7.6); H4: 8.69 (t, 7.6). $^{13}C$ NMR: $C_2H_2$: 59.7 [J($^{13}C^{117/119}Sn$): 40 Hz]; C2 = C6: 145.3; C3 = C5: 126.5; C4: 146.6; C2' = C6': 162.4. $^{119}Sn$ NMR: -166.2 {sep, 1271}.

Compound **3**: 300 mg (0.71 mmol) of $(o\text{-}C_2B_{10}H_{11}\text{-}9)_2SnO$ were added to a solution of 184 mg (1.42 mmol) of 2-L-pyrrolidone-5-carboxylic acid in 50 mL of ethanol/benzene (10/40). The mixture was refluxed during 4 hours and the water/ethanol/toluene ternary azeotrope was distilled off with a Dean-Stark funnel. The remaining solvent was evaporated under reduced pressure. Recrystallisation from hexane/benzene yielded white needles: 79 %; mp 248-251 °C. Mössbauer: IS = 1.62; QS = 3.83; $\Gamma_1$ = 0.90; $\Gamma_2$ = 0.95. NMR solvent: $CDCl_3$. $^1H$ NMR: $C_2H_2$: 3.15 (bs), $B_{10}H_9$: 1.2-3.4 (b); NH: 6.13 (bs); H5: 4.29 (m); H3, H4: 2.2 - 2.6 (m). $^{13}C$ NMR: $C_2H_2$: 58.3 [J($^{13}C^{117/119}Sn$): ca. 40 Hz, broad]; C5: 55.5; $CH_2$: 25.2, 29.5; CO, COOSn: 177.7, 179.5. $^{119}Sn$ NMR: -61.0 {sep, 1230}.

### *Antitumour Tests*

### *In Vitro Tests*

The cytotoxicity of compounds **1** to **3**, together with some reference compounds, was determined *in vitro* against six well characterized human tumor cell lines by applying the microculture sulforhodamine B test (SRB). The compounds were tested in quadruple at 10 concentrations varying with a factor 3, ranging from 3 to 59050 ng/ml.
Concentration response curves were determined and the $ID_{50}$ (drug concentration in ng/mL at 50% growth inhibition) values were calculated.
Prior to the experiments a mycoplasma test was carried out on all cell lines and found to be negative. All cell lines, except EVSA-T, were maintained in a continuous logarithmic culture in

RPMI medium with Hepes and Phenol red supplemented with 10% bovine calf serum (BCS), penicillin 111 IU/ml, streptomycin 111 mg/ml, gentamycin 46 mg/ml and insulin 10.6 mg/ml. EVSA-T was maintained in DMEM with 5% BCS and antibiotics as described. The cells were mildly trypsinized for passage and for use in experiments.
RPMI, DMEM and SRB (sulforhodamine B) were obtained from Brunschwig (Amsterdam, The Netherlands). BCS was obtained from Hyclone (Logan, Utah, USA), DMSO from Baker (Deventer, The Netherlands), phosphate-buffered saline (PBS) from Boom (Meppel, The Netherlands), insulin Neerlandicum from Organon (Oss, The Netherlands). Streptomycin, penicillin, gentamycin and trypsin were obtained from Gibco (Breda, The Netherlands).
The test and reference compounds were dissolved to a concentration of 177147 ng/ml as follows:
Organotin compounds: 1.0 to 2.2 % DMSO in full growth RPMI medium. Carboplatin: 10% water in full growth RPMI medium
Cis-platin: 0.17% DMSO in full growth RPMI medium
No additional pretreatment, as ultra sonication, was needed for complete dissolution of all compounds.
On day 1, 200 ml of trypsinized tumor cells (2000 cells/well) were plated in 96-wells flatbottom microtiter plates (Costar, no. 3799, Badhoevedorp, The Netherlands). The plates were preincubated for 24 hr at 37°C, 5% CO2 to allow the cells to adhere.
On day 2, 100 ml of a solution with the highest drug concentration were added to the wells of column 12 and from there diluted 3-fold to column 3 by serial transfer of 100 ml using an 8 channel micropipette. The final volume of column 3 was adjusted to 200 ml with PBS. Column 2 was used for the blank. PBS was added to column 1 to diminish interfering evaporation.
On day 7 the incubation was terminated by washing the plates twice with PBS. Subsequently the cells were fixed with 10% trichloroacetic acid in Milli Q water (Millipore, Etten Leur, The Netherlands) and placed at 4°C for one hour.
After five washings with tap water, the cells were stained for at least 15 min with 0.4% SRB, dissolved in 1% acetic acid, and subsequently washed with 1% acetic acid to remove the unbound stain. The plates were air dried and the bound protein stain was dissolved by using 150 ml 10 mmol/l tris base. The absorbance was read at 540 nm using an automated microplate reader (Titertec, Flow Laboratories Ltd., Irvine, Scotland).

*In Vivo Tests on DBA/2 mice*
Compound **2** was suspended in 2 % carboxymethylcellulose. The drug was administrated intraperitoneally as a single injection in a volume of 10 mL/kg. On day 0, $1x10^5$ L1210 cells from a stock culture suspension were injected i.p. into male DBA/2 mice. After about 24 hours treatment was started. The drug was administered by a single intraperitoneal injection to groups of 6 mice. Each drug was tested at three dose levels. The highest doses were expected to show toxicity. The untreated control group consisted of 9 mice. The average body weight of the various groups was determined on the day of treatment (day 1), on day 5 and on day 7. The survival time of the mice was recorded. The results are expressed as median survival time (MST) of treated over control mice (T/C %).

**Acknowledgments.**
We thank Mr. H. J. Kolker, Dr. J. Verweij, Prof. Dr. G. Stoter and Dr. J. H. M. Schellens,

Laboratory of Experimental Chemotherapy and Pharmacology, Department of Medical Oncology, Rotterdam Cancer Institute, NL - 3008 AE, Rotterdam, The Netherlands, for having performed the tests.
The financial support from INTAS (Grant 93-659), from the Belgian "Nationale Loterij" (grants 9.0050.90 and 9.0006.93: R. W. & M. B.), from the Belgian "Fonds voor Kollektief Fundamenteel Onderzoek" (grants 2.0127.90: M. G. & R. W. and 2.0094.94; R. W. & M. B.), from the "Ministère des Affaires Culturelles du Luxembourg" (grant number BFR90/036; F. K.), from the "Comité National des Bourses OTAN" (F. K.), from the "Ministère de l'Education Nationale du Luxembourg" (F. K.) and from the "Russian Foundation for Fundamental Research" (grant number 93-03-18654) (V.I.B.) is gratefully acknowledged.

### References

1. G. I. Locher, *Am. J. Roentgenol.* **36**, 1 (1936).
2. R.G. Fairchild and V.P. Bond, *Int. J. Radiat. Oncol. Biol. Phys.* **11**, 831 (1985).
3. M. Gielen, P. Lelieveld, D. de Vos and R. Willem, "In vitro antitumour activity of organotin compounds", in "Metal-Based Antitumour Drugs", Vol. 2, Ed. M. Gielen, Freund Publishing House, Tel Aviv, pp. 29 - 54 (1992); M. Gielen, H. Pan, R. Willem and D. de Vos, Pharmachemie B.V., Eur. Pat. 90202936.2-, 06/11/90, Antitumor compositions and compounds, *Chem. Abs.* **116**, 235 873q (1992); M. Gielen, P. Lelieveld, D. de Vos, Huade Pan, R. Willem, M. Biesemans and H.H. Fiebig, *Inorg. Chim. Acta*, **196**, 115 (1992)
4. M. Gielen and R. Willem, *Anticancer Res.*, **12**, 1323 (1992); M. Gielen and R. Willem, *Anticancer Res.*, **12**, 257 (1992); M. Gielen and R. Willem, *Anticancer Res.*, **12**, 269 (1992)
5. V. I. Bregadze, V. Ts. Kampel and N.N. Godovikov, *J. Organomet. Chem.* **157**, C1 (1978)
6. R. Willem, A. Delmotte, M. Biesemans, I. De Borger, M. Gielen, F. Kayser and E. R. T. Tiekink, *J. Organomet. Chem., in press.*
7. Kennedy, J. D., *"Multinuclear NMR"*, ed. J. Mason, Chapter 8, 221-58.
8. A. G. Davies, P. G. Harrison, J. D. Kennedy, R. J. Puddephatt, T. N. Mitchell and W. McFarlane, *J. Chem. Soc.* **C** 1136 (1969).

**Received: July 4, 1994 - Accepted: August 8, 1994 - Received in camera-ready format: September 15, 1994**

